# Rotational Stiffness and Carrying Capacity of Timber Frame Corners with Dowel Type Connections

**DOI:** 10.3390/ma14237429

**Published:** 2021-12-03

**Authors:** Marek Johanides, David Mikolasek, Antonin Lokaj, Petr Mynarcik, Zuzana Marcalikova, Oldrich Sucharda

**Affiliations:** 1 Department of Structures, Faculty of Civil Engineering, VSB-Technical University of Ostrava, 708 00 Ostrava, Czech Republic; david.mikolasek@vsb.cz (D.M.); antonin.lokaj@vsb.cz (A.L.); zuzana.marcalikova@vsb.cz (Z.M.); 2 Department Centre of Building Experiments, Faculty of Civil Engineering, VSB-Technical University of Ostrava, 708 00 Ostrava, Czech Republic; petr.mynarcik@vsb.cz (P.M.); oldrich.sucharda@vsb.cz (O.S.); 3 Department of Building Materials and Diagnostics of Structures, Faculty of Civil Engineering, VSB-Technical University of Ostrava, 708 00 Ostrava, Czech Republic

**Keywords:** rotational stiffness, wood, timber, frame connection, screws, glued laminated timber, numerical model, finite element method

## Abstract

With the development of wooden structures and buildings, there is a need to research physical and numerical tests of wood-based structures. The presented research is focused on construction and computational approaches for new types of joints to use in wooden structures, particularly glued lamella elements made of wood and wood-based composites. This article focuses on improving the frame connection of a wooden post and a beam with the use of fasteners to ensure better load-bearing capacity and stiffness of the structure. In common practice, bolts or a combination of bolts and pins are used for this type of connection. The aim is to replace these commonly used fasteners with modern ones, namely full thread screws. The aim is also to shorten and simplify the assembly time in order to improve the load-bearing capacity and rigidity of this type of frame connection. Two variations of the experimental test were tested in this research. The first contained bolts and pins as connecting means and the second contained the connecting means of a full threaded screw. Each experiment contained a total of two tests. For a detailed study of the problem, we used a 2D or 3D computational model that models individual components, including fasteners.

## 1. Introduction

Wood is an important building material that can be used in all applications of construction tasks. However, in some respects, it requires specific approaches, such as testing of mechanical properties and classification [1,2]. It is also possible to use specific dynamic testing [3] or the vibration acoustic method [4]. The diagnostics of existing structures and the determination of mechanical properties are closely related to the testing of mechanical properties [5]. The current possibilities of wood processing technology and design of structures allow a wide range of applications from simple structures, through multi-story buildings [6] to long-span structures [7]. However, it is important to take into account the experience from previous accidents and structural failures in design and analysis [8]. For a correct understanding of the behavior and design of wooden structures, it is appropriate to use experimental tests of structural details [9,10,11] or structural parts, such as entire frames [12]. For example, the use of numerical modeling based on the finite element method is also very important [13,14,15]. It is most often used to determine rotational stiffness, overall load capacity, or failure mechanism. It is also possible to simulate dynamic loading [11]. Typical experiments include, in particular, frame corner [16], timber-to-timber joints [17], connection/joint [18], and detail [19] experiments.

In the Czech Republic and Slovakia, materials such as steel and reinforced concrete are used in construction more than wood. Of course, structural timber is also used but mainly for the construction of the roof structure or column structure of a building, for example, with a two by four system, or as formwork or visual elements [20].

Due to these facts, current standards are more focused on carpentry joints and the assessment of rod wooden structures produced from sawn wood. Therefore, no calculations or testing procedures have been developed for large joints or large elements made of glued laminated timber. Many production problems and questions associated with the calculation and assessment of such elements arose with the onset of the production of dimensional elements from glued laminated timber. New types of joints and dimensions of the structure brought new types of stresses that older standards for structural design did not identify.

Thanks to modern computer technology, mathematical models, and physical testing of structures on a real scale, it is now possible to design new types of structures, including their connections. Numerical modeling is a valuable and necessary tool in examining the nature of the structure as a whole; moreover, it enables clarification of the nature of various details of the structure, especially joints. It is possible to better understand the interaction between the material and the geometry of the structure by correctly designing and setting the numerical model. With the correct numerical model, we can save a lot of time, energy, and resources when designing the structure and details.

Today, with the emphasis on ecology and renewable resources, wooden structures have begun to appear in our region. Wood is a material with a high variability of properties and the only renewable raw material that comes from nature and disappears without a negative impact on the environment. Information on wood from the Czech Republic can be found in [21]. The most influential factors that cause a variance in wood properties include the type of wood and the locality in which the wood grew. Other influences on the properties of wood are the soil, climate, altitude, the season in which the tree was cut, and, last but not least, the method and quality of processing. In the case of wood as a construction material, we speak either of sawn wood, which is obtained by cutting from conifers or broad-leaved trees [22], or wood-based materials, such as glued laminated timber [23], which is produced by gluing wooden squares to the required size and shape. Wood shows different physical and mechanical properties in mutually perpendicular directions. This means that the properties observed parallel to the fibers are different from the properties observed perpendicular to the fibers. Wood has the greatest strength and stiffness and the least deformation due to moisture and temperature in the direction parallel to the fibers. The mechanical properties of wood define its ability to withstand external loads. In this context, it is necessary to distinguish between the properties of class A wood and structural timber. The properties of class A wood show a relatively large diffusion, which intensifies the most in the case of structural timber due to the influence of growth inhomogeneities. In terms of mechanical properties, wood is an anisotropic material, but for calculations or numerical modeling, it can be considered a material of rectangular or cylindrical orthotropy [21].

One of the uses of wood, among others, is the construction of hall buildings, which are very popular due to their design; moreover, they are of fundamental importance in the construction industry. In addition to the mentioned factors in the selection of construction material for indoor buildings, there is also a fundamental emphasis on the use of natural and aesthetic materials [24,25,26]. This is typical, for example, for the construction of sports facilities. For these reasons, wooden structures are being increasingly used for construction.

The designer cannot normally experimentally test a design. This is mainly due to the financial and time-consuming nature of the experiment. Therefore, the creation of a numerical model or analytical model based on the recommendations of applicable standards and academic literature is common for a design. In practice, however, selecting the wrong connection often leads to oversizing of structural elements, and thus, to an uneconomical design of the building.

The frame connection of these structural elements [27,28] is most often used between the beam and the post. Such connection of elements is one of the most important areas for the designing of wooden structures; the issue of design and assessment of joints of wooden structures fundamentally affects the overall composition of the supporting structure and the dimensions of the main supporting elements [29]. By optimizing this point, significant material savings can be achieved during construction, which will reduce the cost and complexity of the construction.

The load-bearing capacity and stiffness of joints are often a crucial factor for the design and operation of the structure as a whole, especially in structures with larger range, where the joint is heavily stressed. Joints of wooden frame elements can be addressed in several ways. One can use glued joints [30,31,32], for example, by means of glued steel bars. Another possibility is to form a frame connection from a post, which is arranged in a V-shape [33]. The most commonly used type of joint is the creation of a frame connection between a post and a beam by means of pin-type mechanical fasteners [34].

The subject of this paper is experiments focused on the frame connection of the post and the beam created by means of pin-type metal mechanical fasteners. Modern high-strength screws are currently also used as a semi-rigid coupling means for wood–concrete ceilings, as an alternative to fastening with a glued steel bar [35]. Correctly evaluating the behavior and load-bearing capacity of timber connections is important, for example, to evaluate structural timber connections with different distances between the fastener and the loaded end at different moisture contents [36]. The aim of this paper was also to create a more durable connection to the same structure, so the geometry, cross-section of structural elements, and the material of the strut and partition of both experiments were identical. Two types of experiments were created, each containing two tests. The connection was made in the first experiment from the standard mechanical pin-type fasteners that we encounter in common practice. This was a combination of bolts and pins. In the second experiment, the frame connection of the post and the beam was made of full threaded screws, which are not used for this type of connection in common practice. Therefore, the motivation was to analyze in detail this type of connection to determine how the structure functions as a whole, as well as the load-bearing capacity of the frame joint and the rotational stiffness, important for the redistribution of internal forces in the structural system of the bar model.

There is currently no standard for determining the load-bearing capacity and rotational stiffness of frame joints. Therefore, the analytical determination of these values was based on academic literature and articles [37,38], from which the bearing capacity and rotational stiffness of the joint were calculated. In addition, the translational stiffness and load-bearing capacity of the bonding agent were calculated from the standard [39] and current approaches to the design of wooden structures [40,41].

## 2. Materials and Methods

### 2.1. Description of Construction and Geometry

**Figure 1:**
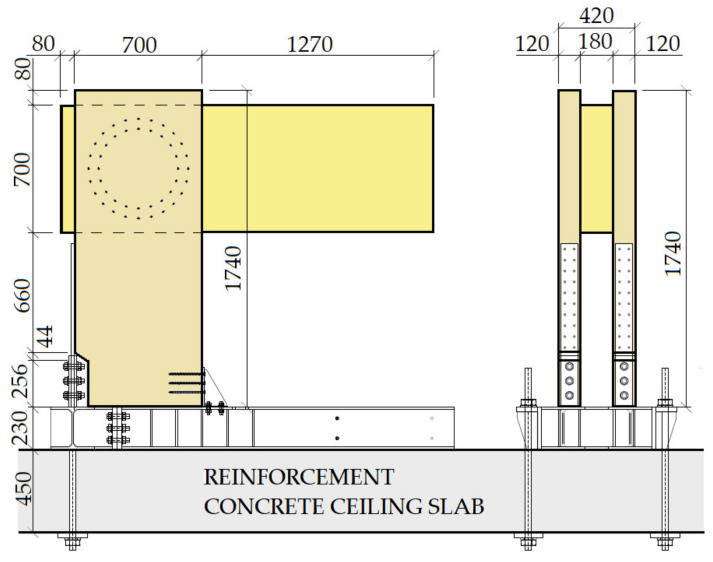
Design test system.

The construction system of the experiment consisted of a bending rigid connection of the frame post and the beam, which was formed from a metal mechanical connecting means of the pin type. The frame post had a cross section of 180/700 mm, with a wood class of GL24h. The frame beam had a cross section of 2 × 120/700 mm, wood class GL24h. The geometry and dimensions of the structural elements of the experimental tests were identical; the difference can be found in the connecting means, the number, and the geometries of the arrangement. The structural elements were connected by a bending rigid connection by means of a combination of bolts and pins with a diameter of 12 mm in experiment setup no. 1 (test no. 2 and test no. 4). In experiment setup no. 2 (test no. 1 and test no. 3), this connection consisted of an 11 mm diameter full thread screw. In order to perform this experiment, it was necessary to create the rigid boundary conditions of the structure. To ensure the correct boundary conditions, a steel structure was made, which was anchored by means of a threaded rod into a reinforced concrete ceiling slab 450 mm thick. The posts of the frame structure were attached to this structure by means of steel sheets and screws, which ensured the boundary conditions. The geometry of the frame corner structure and a sample of the steel structure are shown in Figure 1. The individual frame connections are described in the section below.

### 2.2. Experiment Setup No. 1—Joint Created from a Combination of Bolts and Pins

**Figure 2:**
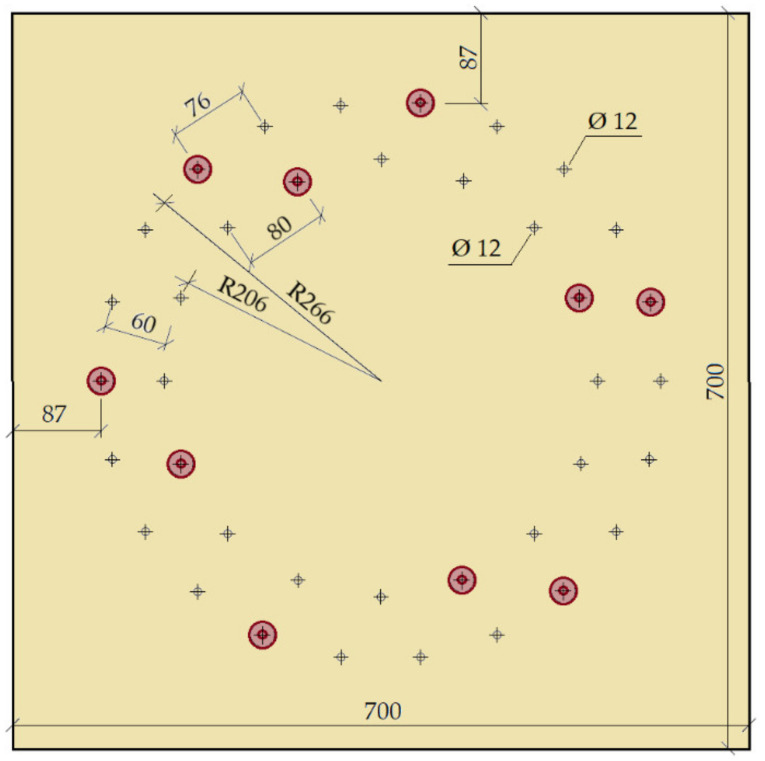
Location of fasteners, bolts, and pins (experiment setup no. 1).

This experiment consisted of creating a frame connection between the post and the beam using bolts and pins with a diameter of 12 mm. The material of the fasteners was steel of class 8.8. The arrangement of the bolts and pins was on two symmetrically centered circles. Circle 1 had radius *r*_1_ = 266 mm, and there were 22 fasteners on it. Circle 2 had radius *r*_2_ = 206 mm, and there were 16 fasteners on it. The joint geometry and detailed arrangement of the screws is shown in Figure 2.

The arrangement of the fasteners from the loaded/unloaded edge and between the fasteners was not defined in [40] for a circular arrangement. Therefore, these distances were determined on the basis of recommendations given in the literature [41].

The ratio between the number of bolts and pins was chosen based on the recommendations of the literature [41].

### 2.3. Experiment Setup No. 2—Joint Created from Full Threaded Screws

**Figure 3:**
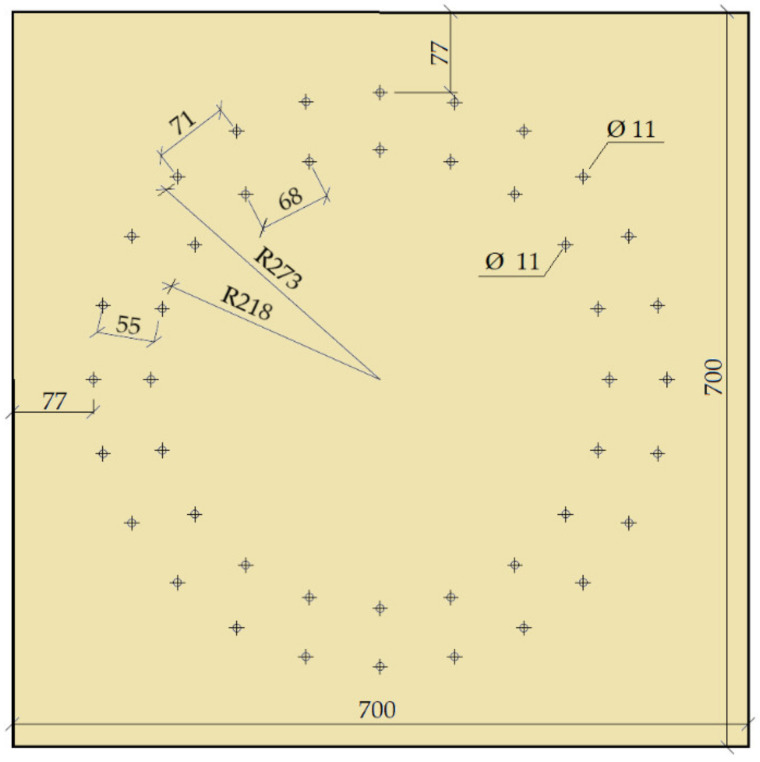
Location of fasteners, and full threaded screws (experiment setup no. 2).

This experiment consisted of creating a frame connection of the post and the beam using full-threaded screws. The diameter was 11 mm, and the length of the screw was 400 mm. The material of the screw was steel class 10.9. The arrangement of the screws was on two symmetrically concentrated circles, where circle 1 had radius *r*_1_ = 273 mm, and there were 24 screws on it. Circle 2 had radius *r*_2_ = 218 mm, and there were 20 screws on it. The joint geometry and detailed arrangement of the screws are shown in Figure 3.

The arrangement of the fasteners from the loaded/unloaded edge and between the fasteners was not defined in [40] for a circular arrangement. Therefore, these distances were determined on the basis of recommendations given in the literature [41].

### 2.4. Description of Sensor Location

**Figure 4:**
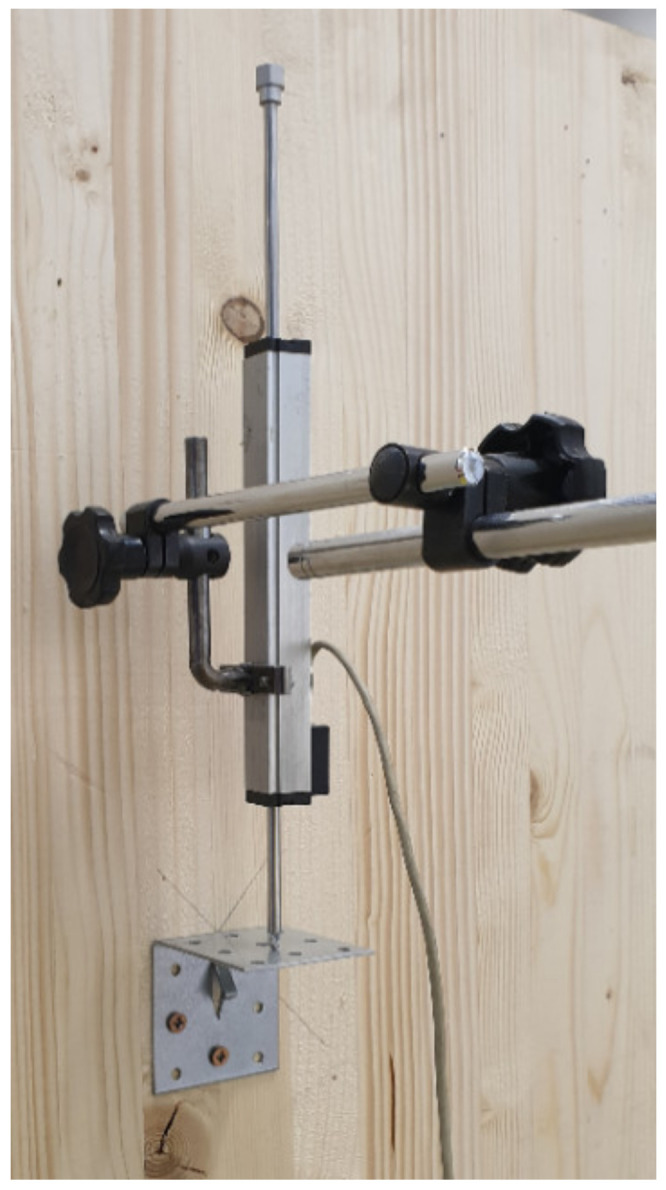
Displacement tracer—sensor from company Ahlborn [42].

Three deformation sensors were mounted on the structure in order to be able to monitor the deformation of the structure, on the basis of which we later determined the value of rotational stiffness. Specifically, they were potentiometer sensors, which belong to the category of resistance sensors, Type TR–0100. The displacement tracer - the spring potentiometer linear position sensor, which had a linear deviation of 0.01 mm with a maximum stroke length of 100 mm, is shown in Figure 4.

**Figure 5:**
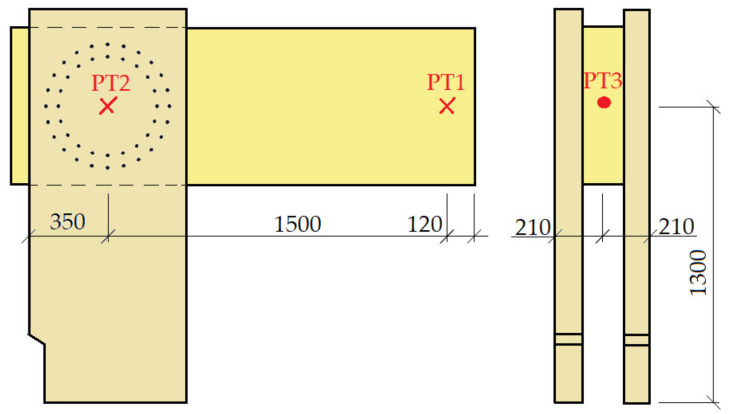
Deformation sensor location display, front view on the left, side view on the right.

Two sensors were placed on the cross-sectional axis of the beam. One was at the end of the free end of the beam, under the load (press head), and the other was placed in the middle of the frame corner (see the cross points in Figure 5). Due to the location of these two deformation sensors, we were able to measure and calculate the actual vertical deviation of the measured points. It was necessary to fit another deformation sensor to the structure. It was mounted on the post, specifically on the imaginary axis of the beam. This sensor recorded the horizontal tilt of the construction (see the full point in Figure 5).

Based on the arrangement of the deformation sensors, we were able to separate the individual components of deformation and tilt; thus, we calculated the actual value of the rotational stiffness of the frame connection of the post and the beam.

### 2.5. Description of Test Equipment

The experiment was performed in the experimental construction center, Faculty of Civil Engineering VSB—Technical University of Ostrava, Czech Republic, on a hydraulic servo roller, which allowed tensile, pressure static, and dynamic tests. The maximum force that the electrohydraulic rollers of the test equipment could generate was 400 kN, which was sufficient for testing the frame system up to failure.

### 2.6. Description of the Loading Process

To perform experiments, it can be noted that the choice of loading (force/deformation) must be chosen individually with respect to a particular experiment (structure/part of the structure). From the point of view of design standards, the force load is more suitable for the linear area of loading. Deformation load, which is usually also used in numerical modeling in nonlinear analysis, is more suitable for determining the overall course of the experiment. It is possible to combine both approaches of loading (force/deformation), but this is suitable for tests that are repeated several times. The Newton Raphson Method and deformation load were used in the nonlinear calculation. Therefore, the same method was chosen for the experiments. The basic/initial process of loading was determined according to the standard in [43]. The structure was first loaded to about 40% of the characteristic load capacity, the value of which was calculated according to the standard in [40] and the professional literature [41]. Subsequently, the construction was lightened. This was followed by a second cycle in which the load was about 70% of the characteristic load capacity. This step was followed by loading until the structure was damaged. Table 1 shows the load values shows the load curve for experiment setup test no. 1 on bolts and pins fasteners, determined according to [44]. Table 2 shows the load values shows the load curve for experiment setup test no. 2 on screw fasteners, determined according to [44]. The resulting loading process is clearly visible in the resulting load–displacement diagrams.

**Figure 6:**
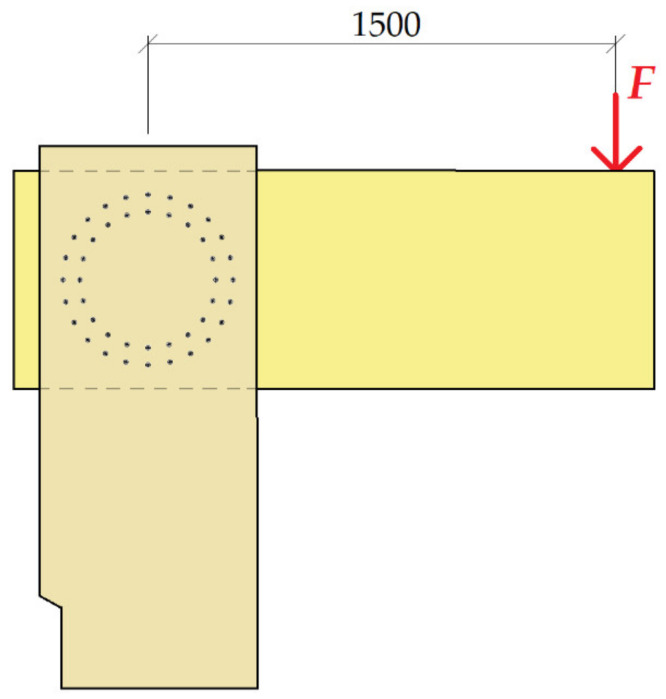
Position of the load *F*.

Figure 6 shows the position of applying the load “*F*” to the structure. The position of loading into the structure was the same for both tests.

### 2.7. Calculation of the Load-Bearing Capacity of the Frame Connection

The literature [41] was used to determine the load-bearing capacity of the frame connection. The calculation of the bearing capacity was based on Johansen’s relations. The frame connection was a double shear connection of the wood–wood type.

(1) $$F_{v,Rk}=min\left\{\begin{array}{c} f_{h,1,k}\cdot t_{1}\cdot d\;\\ 0.5\cdot f_{h,2,k}\cdot t_{2}\cdot d\;\\ 1.05\cdot \frac{f_{h,1,k}\cdot t_{1}\cdot d}{2+\beta }\cdot \left[\sqrt{2\beta \cdot \left(1+\beta \right)+\frac{4\beta \cdot \left(2+\beta \right)\cdot M_{y,Rk}}{f_{h,1,k}\cdot d\cdot t_{1}^{2}}}-\beta \right]+\frac{F_{ax,Rk}}{4}\\ 1.15\cdot \sqrt{\frac{2\cdot \beta }{1+\beta }}\cdot \sqrt{2\cdot M_{y,Rk}\cdot f_{h,1,k}\cdot d}+\frac{F_{ax,Rk}}{4}\;\;\;\;\; \end{array}\right.,$$

with

(2) $$\beta =\frac{f_{h,2,k}}{f_{h,1,k}},$$

where:

*F_v,Rk_* is the characteristic load-bearing capacity of one cut of one fastener;
*t_i_* is the wood thickness 1 or 2;
*d* is the diameter of the fasteners;
*M_y,Rk_* is the characteristic plastic moment of bearing capacity of the fastener;
*β* is the ratio between the compressive strengths of the elements; and
*F_ax,Rk_* is the characteristic axial load capacity for pulling out the fasteners.

### 2.8. Calculation of Translational and Rotational Stiffness of a Frame Connection

To calculate the internal forces, it is important to correctly capture the rigidity of the connections. The main characteristic of the rigidity of the joint of wooden structures is the tempering module *K*_ser_; this value expresses the displacement of the fasteners from a given shear force in the shear surface and the axial force. The torsional spring stiffness *K*_r_ expresses the rotation from the moment. These displacement modules *K*_ser_ were calculated according to the standard in [40], and *K*_r_ was calculated according to literature [41].

### 2.9. Calculation of Translational Stiffness

The relationship applies to one cut of a transversely stressed bolt screw:

(3) $$K_{\mathrm{ser}}=\frac{\rho_{m}{}^{1.5}\cdot d}{23},$$

where:

*ρ*_m_ is the average density of the connected wooden element; and
*d* is the diameter of the fastener.

The literature [40,41] allows the value of *K*_ser_ to be doubled for the steel–wood joint:

(4) $$K_{\mathrm{ser}}=780\cdot d^{0.2}\cdot l_{\mathrm{ef}}{}^{0.4}$$

where:

*l*_ef_ is the effective length of screw penetration; and
*d* is the screw diameter.

The design value of the release module is calculated as

(5) $$K_{u}=\frac{2}{3}\cdot K_{\mathrm{ser}},$$

The translational stiffness of the joint is given by the sum of the slip modules of the individual fasteners. For a two-shear connection with *n* fasteners in ULS, the following relation applies:

(6) $$K_{t,u}=2\cdot n\cdot K_{u},$$

where:

*n* is number of fasteners; and
*K*_u_ is translational stiffness of the fasteners in Relation (5).

### 2.10. Calculation of Rotational Stiffness

The calculation of the torsional spring stiffness *K*_r_ of the flexible joint can be determined by means of the slip module of the fastener in ULS *K*_u_ or the slip module of the fastener in the SLS *K*_ser_ according to the relation

(7) $$K_{r,u}=\sum_{i=1}^{n}K_{u}\cdot r_{i}^{2}\;\mathrm{resp}\;K_{r,\mathrm{ser}}=\sum_{i=1}^{n}K_{\mathrm{ser}}\cdot r_{i}^{2},$$

where:

*K*_u_ and *K*_ser_ are the slip module in ULS and SLS, respectively in Relations (4) and (5).

Thus, for the torsional spring stiffness of a two-shear joint with *n* same fasteners in ULS, the following relation applies (it can also be calculated analogously in SLS):

(8) $$K_{r,u}=2\cdot \left(K_{u}\cdot r_{1}^{2}+K_{u}\cdot r_{2}^{2}+K_{u}\cdot r_{3}^{2}+K_{u}\cdot r_{n}^{2}\right).$$

### 2.11. Numerical Model

**Figure 7:**
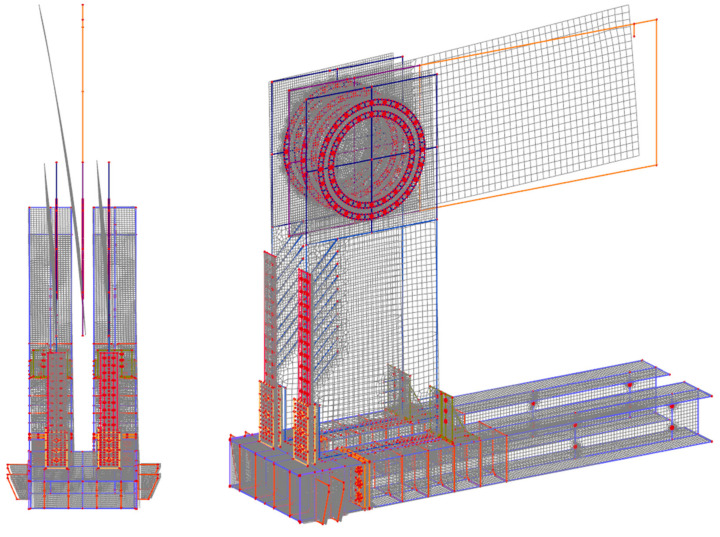
Computational model (global stability)—mesh finite elements.

**Figure 8:**
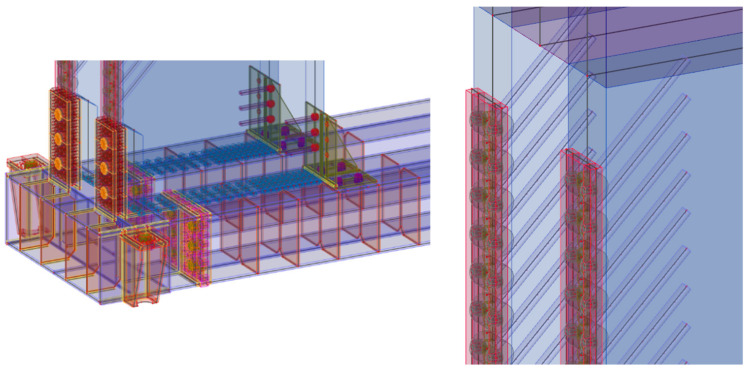
Computational model—detail of the mesh of finite elements of the frame.

**Figure 9:**
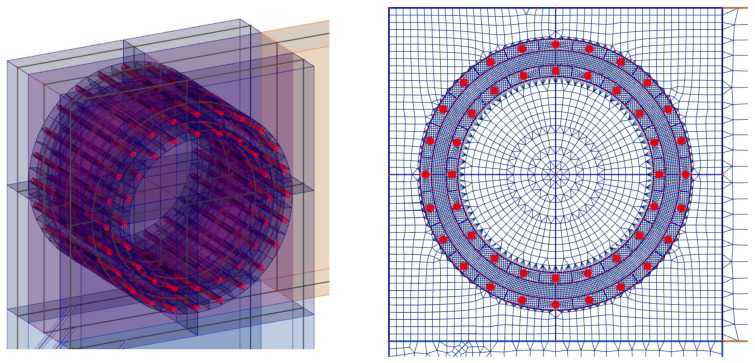
Computational model—detail of the mesh of finite elements of the frame.

**Figure 10:**
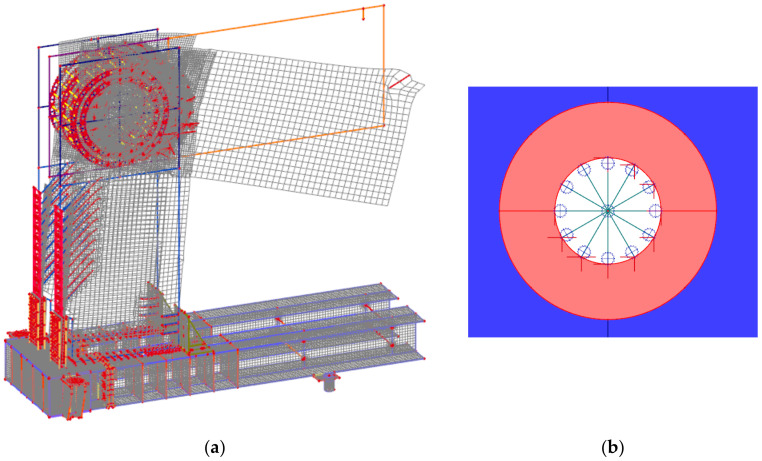
Computational model: (**a**) local stability; (**b**) detail of the mesh of finite elements.

For the experiment of test sample no. 1, two concepts for the computational model were used for numerical modeling. Both approaches to numerical modelling are based on the finite element method. The first concept of numerical modelling is focused on verifying the stability calculation. The computational model consists of shell and beam (rod) finite elements. The detail of the computational model and the finite element mesh is shown in Figure 7. The computational model was processed down to the details of the connecting elements, which are evident in Figure 8 and Figure 9. The connection between the connecting elements and the wood was in the form of stiffening springs (beam), as can be seen in Figure 10b. *SCIA Engineer* software [45] was used for the calculations. The computational model had 82,793 2D finite elements and 3970 1D finite elements. In total, the computational model had 80,277 nodes, and 481,662 equations were solved. The performed calculations were used to verify the stability of the whole system before the experiment.

Calculations of nonlinear stability were performed. Figure 10a shows the local loss of stability. The global loss of stability is shown in Figure 7. The orthotropy of wood was taken into account in the calculations; the input parameters for the element with thickness are given in Table 3. For elements with a different thickness, the procedure was similar using the recommendations of the *SCIA Engineer* software [45]. The force in stability was calculated with a value of 132 kN, which corresponds to ultimate limit state. The first shape (Figure 10a) was 4.41, representing local buckling under force.

**Figure 11:**
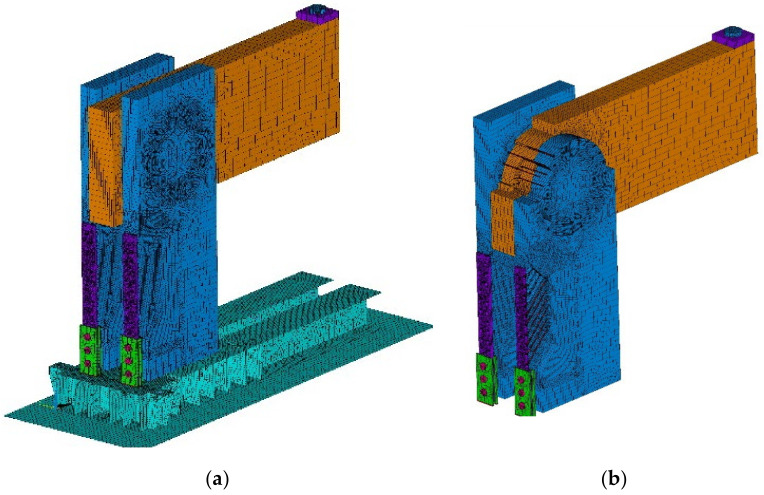
The 3D computational model: (**a**) total model; (**b**) part of the model.

The second chosen concept of numerical modeling is based on the 3D computational model, which is shown in Figure 11. In the computational model, solid, shell, and beam finite elements were used, supplemented by contacts at the interface. *ANSYS*^TM^ software [46] was used for the calculations. The computational model used finite elements: shell181, steel chassis base and frame corner; beam188, eccentricity exchanges and connections; and solid45, timber. Targe170 and conta174 elements were used for contacts. In total, the computational model had 543,939 nodes and 3,026,939 elements, and 1,693,955 equations were solved.

**Figure 12:**
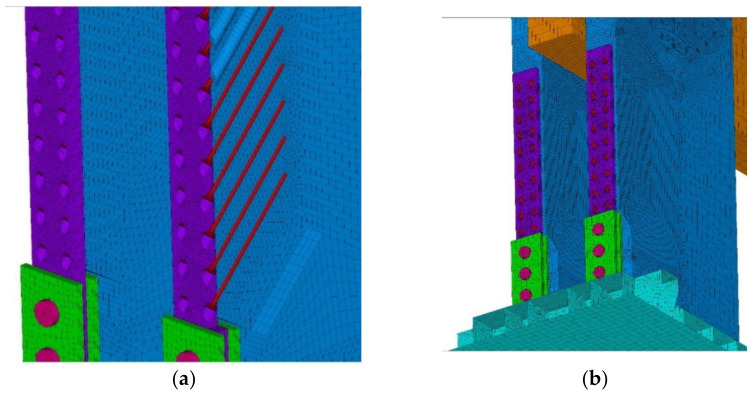
The 3D computational model: (**a**) detail no. 1; (**b**) detail no. 2.

The goal of the computational models was to simulate the behavior during the test experiment. Again, the computational model was processed down to the detail of the fasteners, as shown in Figure 12a,b.

Wood behaves as a strong anisotropic material, but simplification to orthotropic behavior can be considered using numerical methods. When considering orthotropic material, it is possible to use a cylindrical model, which considers the curvature of annual rings, or a rectangular one, which does not consider the curvature of annual rings. We used a numerical orthotropic model, and the effects of annual rings were neglected, namely, the difference between spring and summer wood, local defects of wood such as bumps or cracks, and the variable structure of wood. Table 4 shows elastic constants of the material model of wood in the *ANSYS*^TM^ program [46].

**Figure 13:**
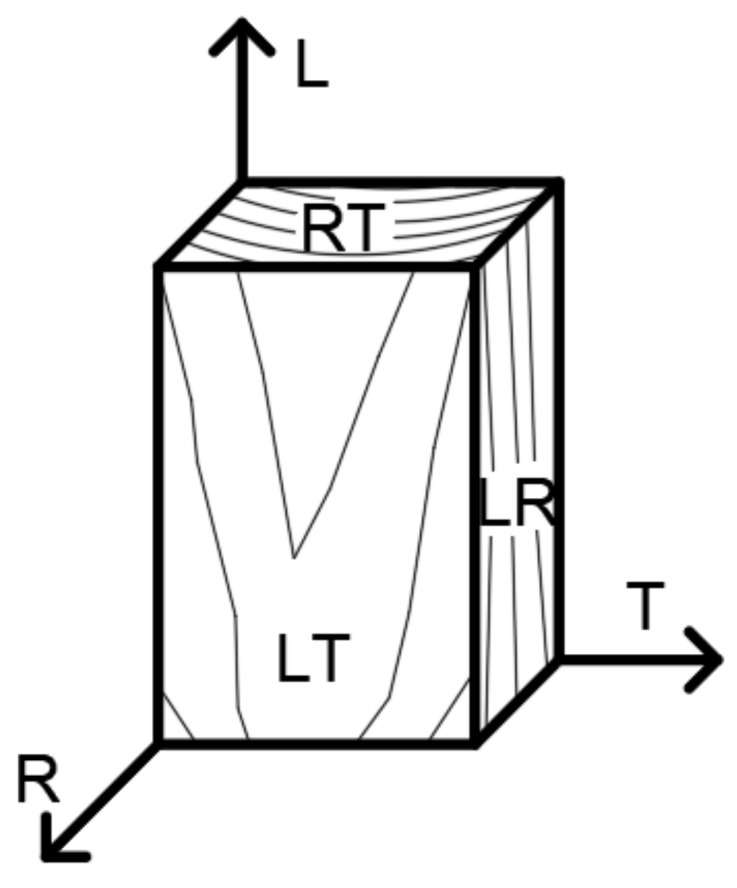
Planes of elastic symmetry of wood.

The material model of wood was defined in the system of rectangular coordinates L, T, and R (see Figure 13) by nine elastic constants, which were the modulus of elasticity in the direction of fibers *E*_L_; modulus of elasticity in tangential direction *E*_T_; modulus of elasticity in radial direction *E*_R_; modulus of elasticity in shear *G*_LT_, *G*_LR_, and *G*_TR_; and the Poisson factors of transverse deformation *ν*_LT_, *ν*_LR_, and *ν*_TR_ (see Figure 13).

**Figure 14:**
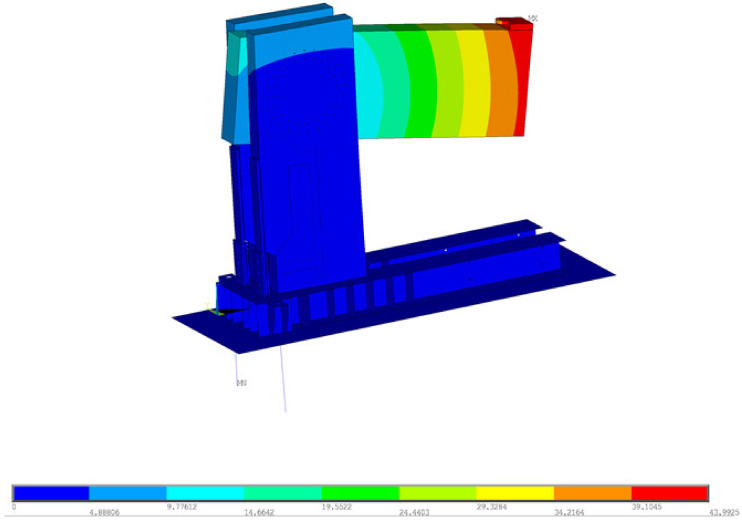
Total deformation of the computational model.

**Figure 15:**
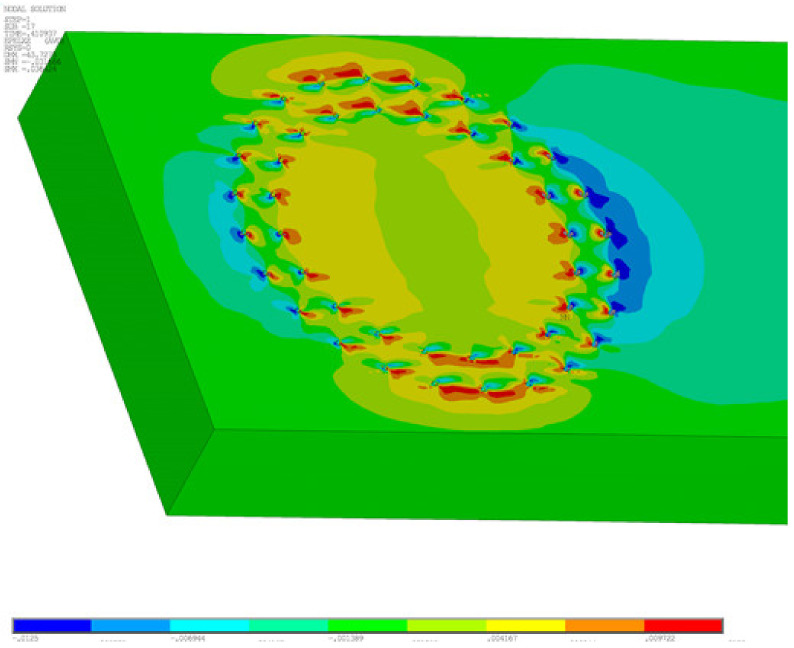
Computational model—strain *ε*_XZ_.

**Figure 16:**
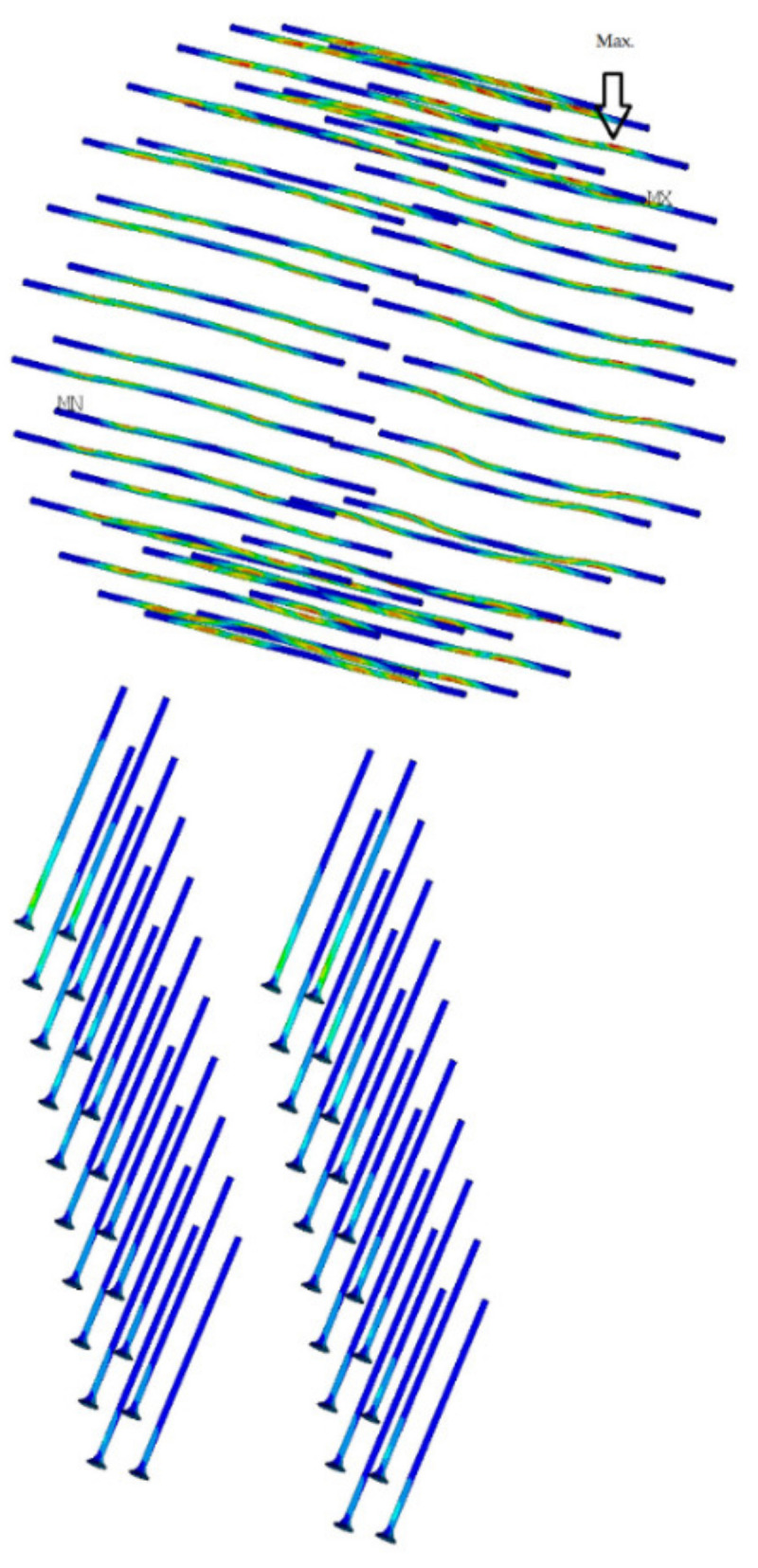
Connecting elements—max. stress.

To illustrate the performed calculations, two graphic outputs were selected from the calculation in Figure 14 and Figure 15. Figure 14 shows the total deformations on a 3D computational model. Figure 15 show the part of the computational with the strain *ε*_XZ_. The 3D computational model allows a detailed study of the stress of the connecting elements; the illustrative results are shown in Figure 16.

### 2.12. Experimental Testing

**Figure 17:**
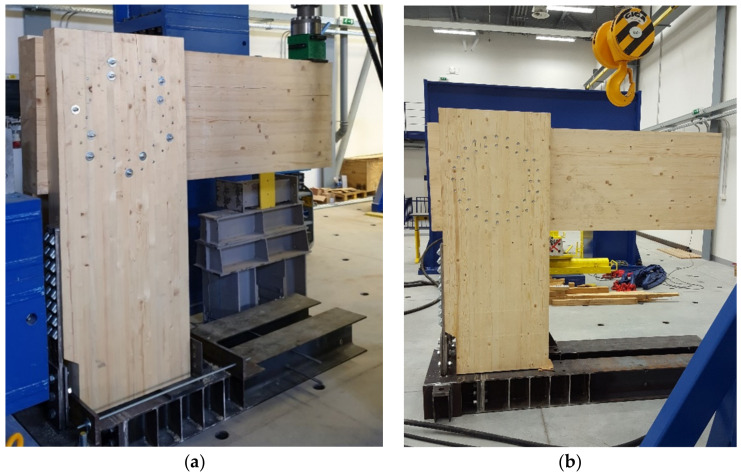
Experimental sample: (**a**) test sample no. 4; (**b**) test sample no. 1.

Individual samples of the frame connection were tested in the experimental construction center, Faculty of Civil Engineering VSB—Technical University of Ostrava, Czech Republic. Figure 17 shows a sample for experimental testing test sample no. 4 and no. 1.

**Figure 18:**
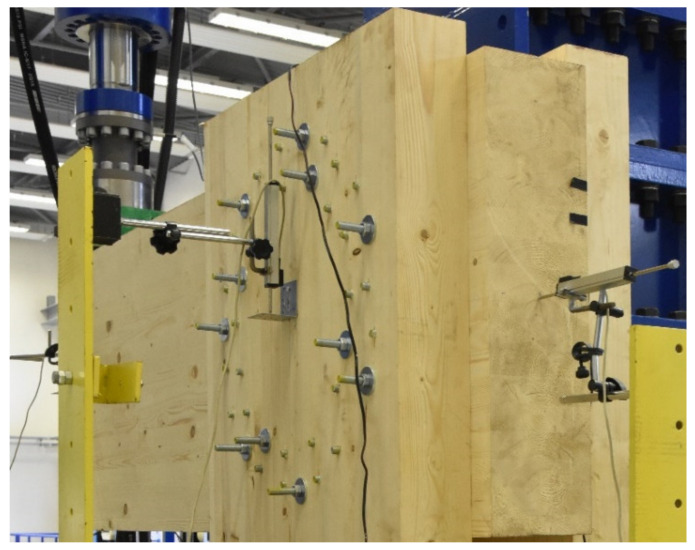
Placement of the deformation sensors.

Figure 18 shows the arrangement of the deformation sensors, according to the diagram shown in Figure 5.

**Figure 19:**
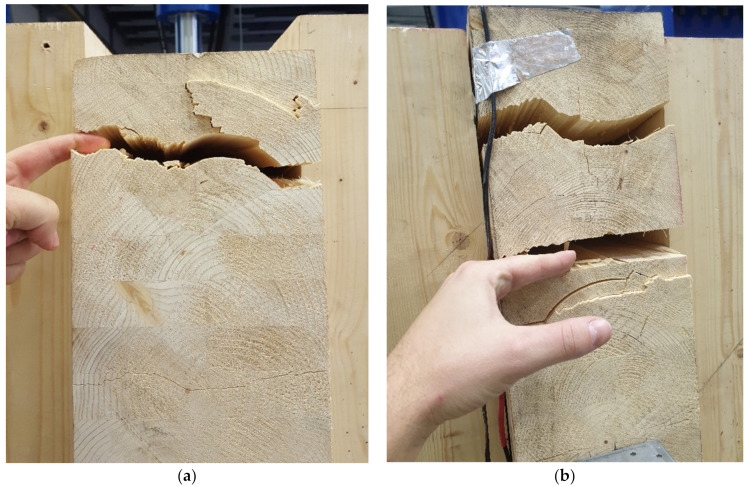
Experiment no. 2: (**a**) damage test sample no. 1; (**b**) damage test sample no. 3.

**Figure 20:**
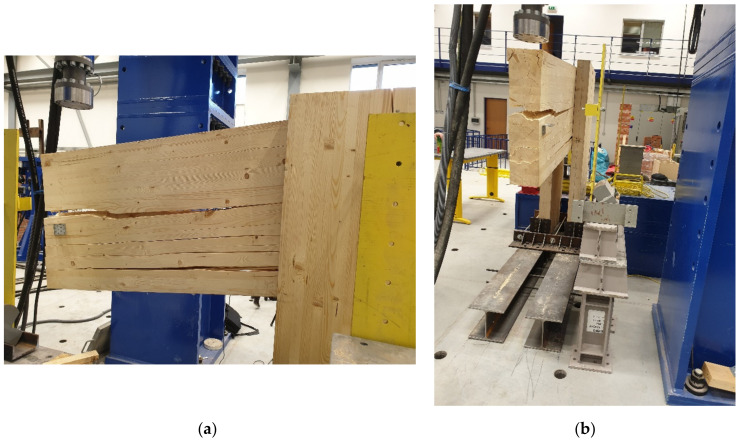
Experiment no. 2: (**a**) damage test sample no. 3; (**b**) damage test sample no. 3.

The following figures show the damage modes of the individual samples. Figure 19 and Figure 20 show the damage of the experimental test sample no. 1 and test sample no. 3, where the frame connections were made of full threaded screws. In these samples, the structure was broken by pulling perpendicular to the fibers at the top of the beam.

**Figure 21:**
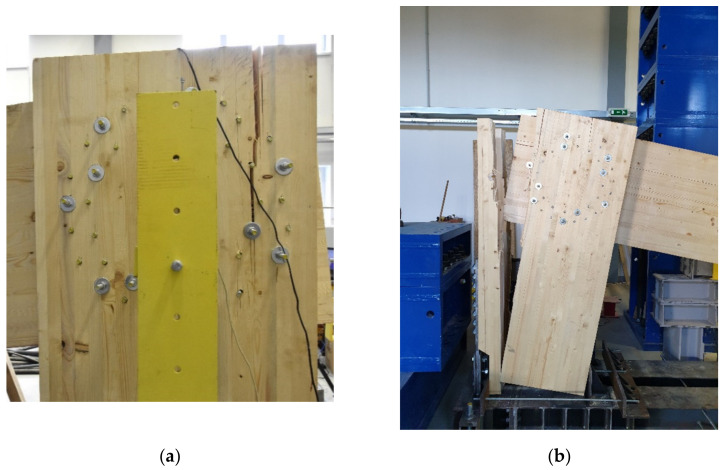
Experiment no. 1: (**a**) damage test sample no. 2; (**b**) damage test sample no. 4.

Figure 21 shows the damage of the experimental testing for test sample no. 2 and test no. 4, where the frame connections were made from a combination of bolts and pins. In these samples, the structure was broken by pulling perpendicular to the fibers in the upper part of the beam, which spread downwards.

## 3. Results

### 3.1. Determination of Bearing Capacity and Rotational Stiffness of Joints for Test Sample No. 1 and No. 3, Full Threaded Screws, on the Basis of Standard and the Literature

The load-bearing capacity of the connection was calculated according to [40,41]. To use the connection to about 100%, a force of 132 kN was used, which was located on the arm 1.50 m (spot of applying force, Figure 6). Table 5 shows the calculation of the load-bearing capacity of the connection for test no. 2 on screws. The table shows the load-bearing capacity of the outer circle, which contained 24 fasteners.

The Relations (3)–(8) were used to calculate the rotational stiffness, and we obtained the value *k*_r_ = 15.03 MNm/rad.

### 3.2. Determination of Bearing Capacity and Rotational Stiffness of Joints for Test Sample No. 2 and No. 4, Bolts and Pins, on the Basis of Standard and Literature

According to [40,41] the load capacity of the connection was calculated. To use the connection to about 100%, the force of 102 kN was used, which was located on the arm 1.50 m (spot of applying force Figure 6). Table 6 shows the calculation of the load-bearing capacity of the connection for test no. 1, bolts and pins. The table shows the load-bearing capacity of the outer circle, which contained 22 fasteners. The fastening effect was neglected in this type of connection.

The Relations (3)–(8) were used to calculate the rotational stiffness, and we obtained the value *k*_r_ = 13.39 MNm/rad.

### 3.3. Comparison of Numerical Model and Experimental Testing

**Figure 22:**
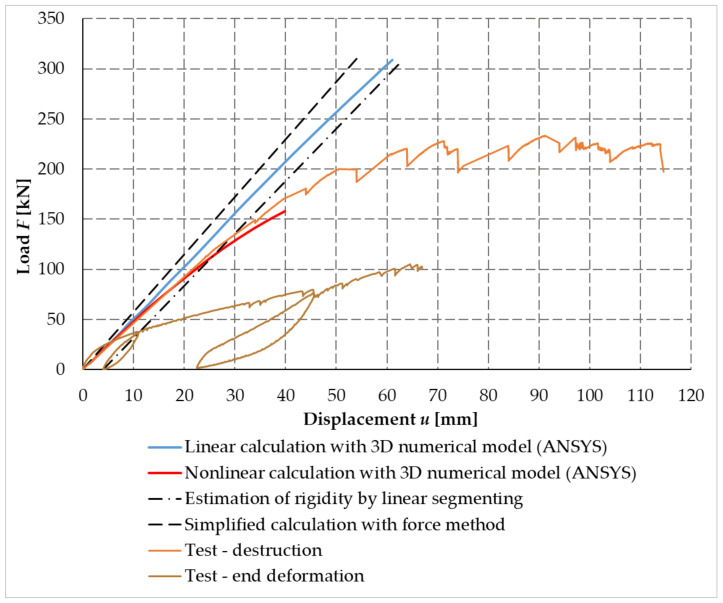
Load–displacement diagram with experiment sample no. 1 and calculations.

Figure 22 shows a load–displacement diagram from the performed testing experiment of sample no. 1 and the calculations performed. Analyzed displacement is at the end of the beam. As part of the testing of the frame corner, two load tests were performed. The first load test marked with a yellow curve included two load steps with relief. The hysteresis of behavior during unloading is clearly visible on the curve. The structure (frame corner) did not collapse during testing. For clarity and comparison, a linearized approximation of the behavior of the tested frame for the elastic loading was performed for the first testing; the black dashed line with dots in the graph denotes the estimation of rigidity by linear segmenting. Furthermore, a second loading of the frame corner was performed, which is marked in orange, when the experiment was terminated by the collapse of the frame corner. The black dashed curve was created according to the analytical relationship of the force method with the simple rod/beam model using the rotational stiffness *K*_r,ser_ = 22.55 MNm/rad (calculated as a characteristic value according to the standard [40]). For the above simplified calculation by the force method, the difference in stiffness with the experiment was the largest. Furthermore, the calculation (blue curve) was performed using a numerical model created in *ANSYS*^TM^ and a linear calculation. The difference between an experiment and a calculation is smaller than for a simplified calculation. A more advanced variant in *ANSYS*^TM^ of the calculation taking into account the nonlinear character of the problem is indicated by a red curve. When comparing the load–displacement curve testing of a numerical model with a nonlinear solution, it can be stated that there was a relatively good agreement between the course and rotational stiffness.

The individual research results (simplified calculation, numerical finite element models, and experimental testing) were compared with each other using some parameters, such as the collapse force of the wooden frame structure and deformation.

Testing also aimed to determine the mode of failure and the force causing the frame connection to collapse. Based on numerical and analytical calculations, it was assumed that the failure was the transverse tensile failure perpendicular to the fibers. This hypothesis was confirmed in experimental testing.

The corner connection of the frame was broken in tensile perpendicular to the fibers in the upper part of the beam at a force *F* = 150.40 kN. It is possible to see the damage of the frame corner face in the pull perpendicular to the fibers in Figure 19.

The values of the maximum loading force of the experimental test were compared with the calculation methods given in Table 7. The value of the calculation according to [40] represented the force at the load-bearing capacity limit for the given frame connection. This value of [40] represented 1.14 times greater resistance than the load-bearing capacity based on experimental testing, showing relatively good agreement of the results. The highest load value came from a linear numerical model created in the *ANSYS*^TM^ program; however, it was not confirmed in experimental testing. On the contrary, the best agreement was represented by a nonlinear numerical model in the *ANSYS*^TM^ program, whose value of the failure force was closest to the force from the experimental testing. This agreement also suggested a fairly accurate numerical model.

### 3.4. Determination of Bearing Capacity Based on Experimental Testing

As already mentioned in the introduction, a total of four tests were created for experimental testing. Two frame connection structures were made of bolts and pins (test sample no. 2 and test sample no. 4) and the remaining two were made of screws (test sample no. 1 and test sample no. 3).

The maximum load-bearing capacities of the frame connection structure were found based on experimental testing, listed in Table 8. This table also shows the calculated load capacities from the analytical solution and these load capacities were mutually divided to obtain the load capacity ratio in the last column.

**Figure 23:**
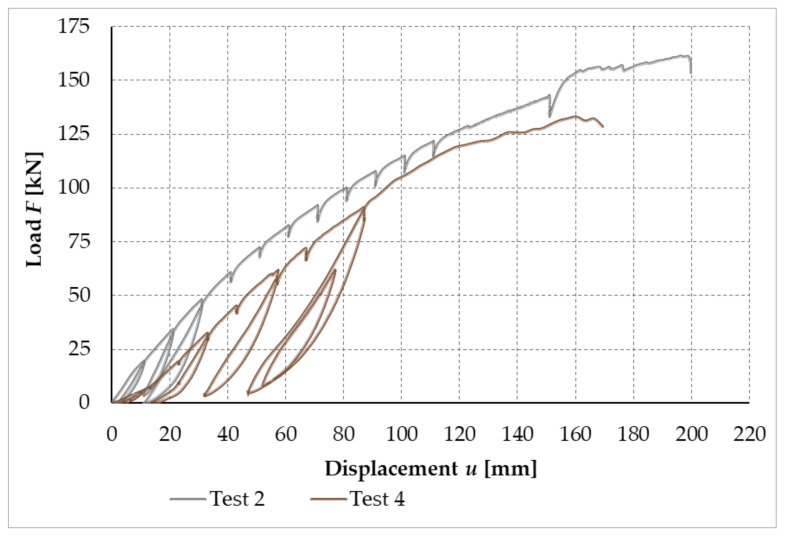
Load–displacement curves of experimental testing, test sample no. 2 and test sample no. 4 (experiment setup no. 1).

Figure 23 shows the working curves of the experimental testing, which illustrate the dependence between the applied load and the deformation of the free end in the frame connection formed from bolts and pins for test sample no. 2 and test sample no. 4.

**Figure 24:**
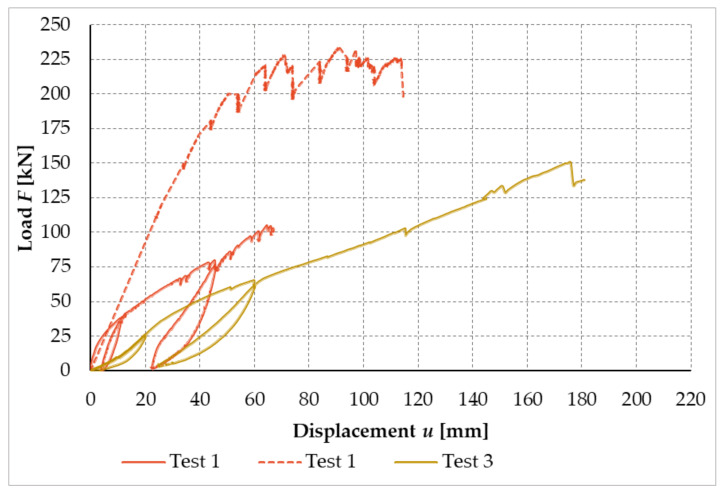
Load–displacement curves of experimental testing, test sample no. 1 and test sample no. 3 (experiment setup no. 2).

Figure 24 shows the working curves of the experimental testing, which illustrate the dependence between the applied load and the deformation of the free end in the frame connection formed from full-threaded screws for test sample no. 1 and test sample no. 3. Test sample no. 1 consists of two working curves. This is due to the fact that during the experimental measurement, it was necessary to readjust the path sensors at a force of about 100 kN.

**Figure 25:**
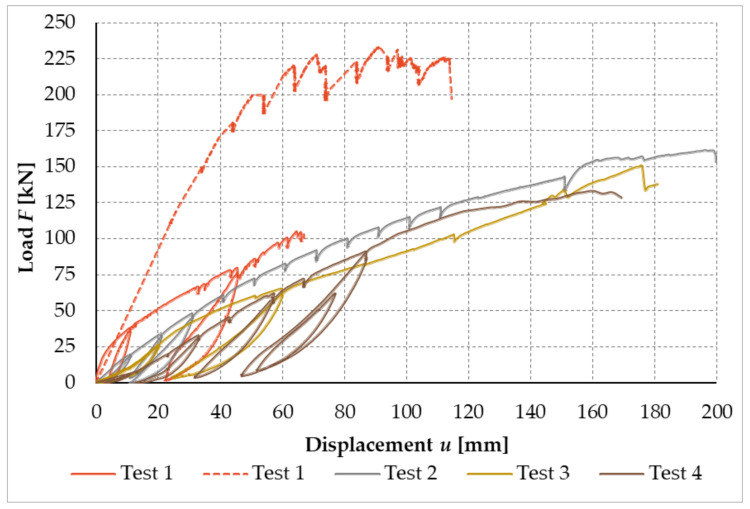
Load–displacement curves of experimental sample tests.

Figure 25 shows the load–displacement curves of all performed experimental tests.

### 3.5. Determination of Rotational Stiffness Based on Experimental Testing

Firstly, it was necessary to calculate all the experimental variables to determine the rotational stiffness of the frame joint. The variables were primarily detected as vertical deformations based on the applied load, from deformation sensors PT1 and PT2. When subtracting these two sensors, we found the actual vertical displacement of the scanned points. However, the section between these points was not a segment but a curve, the path of which was determined by the deformation of the cross-section of the beam by the bending moment and the shear force. Therefore, this value had to be calculated and brought into the calculation of the rotational stiffness of the joint. The vertical displacement of the points, under the sensors PT1 and PT2, was also influenced by the horizontal inclination of the structure due to the load. A deformation sensor PT3 was placed to measure this displacement. The last component that needed to be separated to calculate the rotational stiffness was the deformation of the cross-section of the post, which was caused by the bending moment and the shear force. For the location of said sensors, see Figure 5. After finding all the necessary variable deformations, it was possible to perform the separation of the rotational stiffnesses and thus, to calculate the actual value of the rotational stiffness of the fasteners in the frame connection.

**Figure 26:**
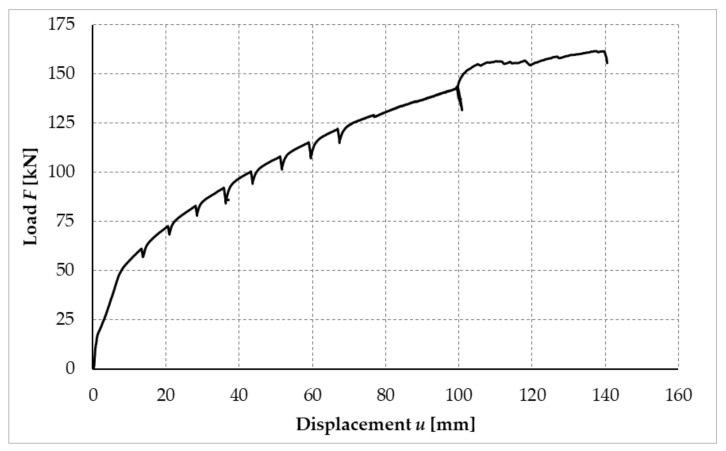
Deformation of the frame joint after separation of deformation components, test sample no. 2.

Figure 26 shows the deformation value of the frame joint after subtracting all the mentioned deformation components. Based on the deformation components separated in this way, it was possible to determine the actual value of the rotational stiffness of the frame joint.

**Figure 27:**
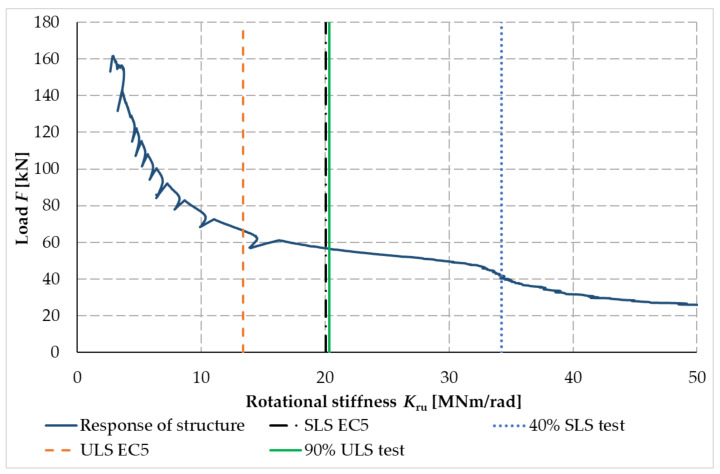
Process of rotational stiffness of frame connection, test sample no. 2.

Figure 27 shows the calculated process of the rotational stiffness of the fasteners. The blue solid curve shown in Figure 27 is the course of rotational stiffness, the value of which is influenced by the magnitude of the applied load. The orange dashed line represents the value of rotational stiffness calculated for the ultimate limit (ULS) state according to [40,41]. The green solid line shows the actual measured value for 90% of the ultimate limit state (ULS) load capacity limit value. The black dashed line with one dot shows the calculated value for the serviceability limit state (SLS) and the blue dotted line shows the actual rotational stiffness value measured for 40% of the SLS load value for the serviceability limit state.

### 3.6. Measured Rotational Stiffness from Experimental Testing

Due to the fact that the PT3 deformation sensor was not fitted when performing test sample no. 1, it was not possible to determine the rotational stiffness of this test.

Table 9 shows the calculated values of rotational stiffness according to [40,41] and the actual measured values of rotational stiffness, which were measured and calculated for test sample no. 2 (bolts, pins) and test sample no. 3 (full thread screws).

The values given in Table 9 were determined based on [40], which recommends determining the value of rotational stiffness for the limit state of serviceability at a load of approximately 40% and for the limit state of ultimate at a load of approximately 90%.

Based on the values given in Table 9, the joint formed from the screws appears to be more rigid compared to the stiffness ratios between the measured values and the values calculated according to [40].

### 3.7. Graphical Comparison of the Results of Numerical Modeling and Experimental Testing

**Figure 28:**
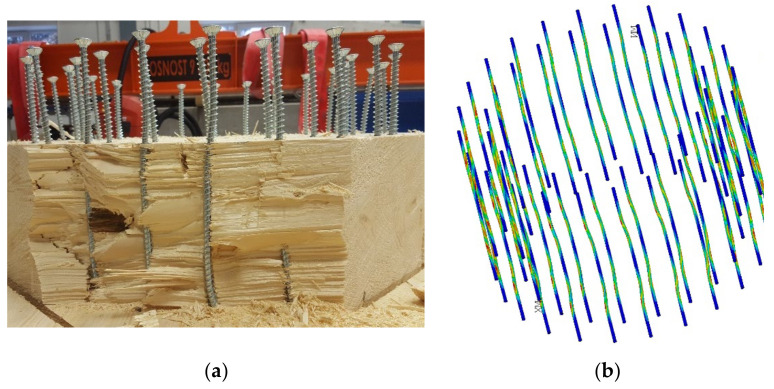
(**a**) Deformed fasteners after experimental testing. (**b**) Deformed fasteners in numerical model *ANSYS*^TM^.

**Figure 29:**
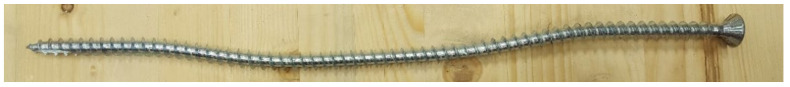
Deformed fastener after experimental testing.

Figure 28 shows the deformed fasteners from the numerical model created in the *ANSYS*^TM^ software and the experimental testing. Figure 29 shows these deformed fasteners in detail after the end of the experimental testing.

## 4. Discussion

This article focuses on the issue of a flexural rigid connection of wooden beams and posts connected by means of pin-type mechanical fasteners. Specifically, two samples were made with full threaded screws and two with bolts and pins. The task required the creation of numerical models and analytical assumptions, which were the basis for the design of experimental tests and were then used to compare the results.

Experimental testing has shown that screws, which are not commonly used to make this type of frame connection, have sufficient load-bearing capacity and rotational stiffness for the purpose of use as standard bolt-and-pin mechanical means.

In addition to the good agreement of the rotational stiffness of the sample with the full-threaded screws, such a connection also provides a load-bearing capacity with a certain margin in comparison with the determination of the load-bearing capacity according to the standard in [41,44] and the literature [40]. Such an assumption was considered because the standards assume a certain margin of bearing capacity before the collapse of a structural element or joint.

Experimental testing of samples formed from bolts and pins confirmed the safety and reliability of the use of these standardly used fasteners [40]. These samples showed a slightly higher value of the ratio between the result of bearing capacity and rotational stiffness of experimental testing and calculation according to standard [41,44] compared to samples formed from full threaded screws.

Frame connections formed of full threaded screws were broken during testing by pulling perpendicular to the fibers at the top of the beam (see Figure 19). This type of damage is predetermined and described in the literature [40] on analytical calculations and, which supports the correctness of the created numerical model.

Frame connections formed of bolts and pins were broken during testing by pulling perpendicular to the fibers at the top of the post (see Figure 21). This different type of damage, compared to samples with full threaded bolts, could occur because bolt and pin fasteners have only a minimal effect of closing the structural elements. Therefore, the stress was concentrated in the cross-section of the post. Damage occurred in it because the cross-section of the post is smaller than the beam. However, in order to verify such a hypothesis, it would be necessary to perform additional experimental tests of identical dimensions and connections.

The tested structure was not reinforced at the damage points, for example, by the use of screws or other additional reinforcements. It is possible to expect an increased escalation in load-bearing capacity by such reinforcement, as determined by the literature [41]. However, in order to verify this hypothesis, it would be necessary to perform experimental tests of samples that would be amplified in this way.

In addition to examining the possibility of using full-threaded screws to create a frame connection in terms of load-bearing capacity and reliability, it is possible to state the following based on these experiments. The frame connection made of full-threaded screws was approximately 1.29 times more durable and approximately 1.10 times stiffer than the connection made on the same surface of the frame connection from bolts and pins. In terms of the diameter and arrangement of bolts and pins, several alternatives were investigated before choosing such a number. The higher load-bearing capacity of the full-threaded screw connection could also save wood mass. This would be an excellent benefit of this connection, as it would save material and money. Moreover, there would be easier manipulation of structural elements, and, last but not least, it would reduce the environmental impact. To confirm this idea, it would be necessary to create further experimental samples having the same diameter and arrangement of the fasteners on the same surface of the frame connection. Therefore, the continuation of experimental measurements is in progress, in cooperation with practice and research activities at the Faculty of Civil Engineering VSB—Technical University of Ostrava, Czech Republic. In particular, experimental measurements of reduced frame connections are underway. The connections are also made of full-thread screws and bolts and pins. The aim of these experimental tests is to support and disseminate the results presented in this article.

## 5. Conclusions

Experimental testing is the most concise way to verify the structural details of wooden structures. It is possible to obtain sufficiently complex knowledge about the action of the joint at a certain nature of the load and the arrangement of the joint in terms of materials, geometry, and design by load tests. The issue of determining the load-bearing capacity of joints of wooden structures according to European standards for the design of wooden structures [41] is constantly evolving. Our experiments, which were aimed at determining the load-bearing capacity and rotational stiffness of the frame connection of a wooden beam and a post using mechanical pin-type fasteners, should also contribute to this trend.

Our experiments have demonstrated the suitability of using high-strength full-thread bolts in the frame connection of the cross member and the strut. The experiments have also demonstrated the reliability and safety of using bolts and pins as fasteners to create a frame connection. In terms of load-bearing capacity, all tested experiments showed a higher load-bearing capacity than the assumption for the ultimate limit state [41]; the connections show safety and reliability with a certain margin. The data of experiments can be advantageously used to calculate rotational stiffness for the purpose of calculating numerical bar models. For a detailed study of the problem, it is also appropriate to use a 2D or 3D computational model that models individual components, including fasteners. The use of experiments can then be applied to 3D numerical modelling using an analysis that takes into account the orthotropic properties of wood. The created computational model enables detailed study of the construction details of pin-type fasteners. The performed calculations sufficiently simulated the plastic behavior of the fasteners.

## Figures and Tables

**Table 1:** The course of test of the experiment setup no. 1, bolts and pins.

Loading Step	From (kN)	To (kN)
Step 1	0 = 0	0.40 × *F*_test_ = 40.80
Step 2	Hold	
Step 3	0.40 × *F*_test_ = 40.80	0.10 × *F*_test_ = 10.20
Step 4	Hold	
Step 5	0.10 × *F*_test_ = 10.20	0.70 × *F*_test_ = 71.40
Step 6	0.70 × *F*_test_ = 71.40	*F*_test_ = 102.00

**Table 2:** The course of test of the experiment setup no. 2, full threaded screws.

Loading Step	From (kN)	To (kN)
Step 1	0 = 0	0.40 × *F*_test_ = 52.80
Step 2	Hold	
Step 3	0.40 × *F*_test_ = 52.80	0.10 × *F*_test_ = 13.20
Step 4	Hold	
Step 5	0.10 × *F*_test_ = 13.20	0.70 × *F*_test_ = 92.40
Step 6	0.70 × *F*_test_ = 92.40	*F*_test_ = 132.00

**Table 3:** Input parameters of wood elements.

Element Width	Modulus of Elasticity of Wood		Modulus of Elasticity in Torsion			Poisson’s Ratio	
	Parallel to the Fibers	Perpendicular to the Fibers					
*h*	*E*_||_	*E*_┴_	*G*_13_	*G*_23_	*G*_12_	*ν*_12_	*ν*_21_
(m)	(GPa)		(GPa)			(-)	
0.12	11	1	0.7	0.7	1.5	0.34	0.031
0.18	11	1	0.7	0.7	1.5	0.34	0.031

**Table 4:** Elastic constants of the material model of wood in the *ANSYS*^TM^ program.

Orthotropic Elasticity	Value	Units
Young’s Modulus *X* direction	11,000	MPa
Young’s Modulus *Y* direction	821	MPa
Young’s Modulus *Z* direction	455	MPa
Poisson’s Ratio *XY*	0.44	-
Poisson’s Ratio *YZ*	0.57	-
Poisson’s Ratio *XZ*	0.56	-
Shear Modulus *XY*	555	MPa
Shear Modulus *YZ*	500	MPa
Shear Modulus *XZ*	677	MPa

**Table 5:** Results of calculation of the characteristic load and capacity of the connection according to [40,41] for test sample no. 1 and test sample no. 3, full threaded screws.

*N*	α	*r*	*x*	*y*	*(g)*	*(h)*	*(j)*	*(k)*	*F*_ax_	*F*_v.Rk_	*F*_v.E_	Use
19	270.00	273.00	−273.00	0.00	38.64	20.57	13.11	6.06	5.32	22.77	22.73	99.79

*α* is the angle between the force and the direction of the fibers; *r* is the radius of the circle where the fasteners are located; *x* is the horizontal coordinate; *y* is the vertical coordinate; (*g*) is Johansen’s relation (*g*)—Relation (1); (*h*) is Johansen’s relation (*h*)—Relation (1); (*j*) is Johansen’s relation (*j*)—Relation (1); (*k*) is Johansen’s relation (*k*)—Relation (1); *F*_ax_ is the switching effect according to [41]; *F*_v,Rk_ is the characteristic load-bearing capacity of one cut of one fastener, Relation (1); *F*_v,E_ is a force acting on one cut of one fastener; and “Use” is the percentage utilization of the connection.

**Table 6:** Results of calculation of the characteristic capacity of the connection according to [40,41] for test sample no. 2 and sample no. 4, bolts and pins.

*N*	α	*r*	*x*	*Y*	(g)	(*h*)	(*j*)	(*k*)	*F*_ax_	*F*_v,Rk_	*F*_v,E_	Use
18	278.18	266.00	−263.29	37.85	39.71	20.37	14.13	10.44	1.08	20.89	20.88	99.95

*α* is the angle between the force and the direction of the fibers; *r* is the radius of the circle where the fasteners are located; x is the horizontal coordinate; *y* is the vertical coordinate; (*g*) is Johansen’s relation (*g*)—Relation (1); (*h*) is Johansen’s relation (*h*)—Relation (1); (*j*) is Johansen’s relation (*j*)—Relation (1); (*k*) is Johansen’s relation (*k*)—Relation (1); *F*_ax_ is the switching effect according to [41]; *F*_v,Rk_ is the characteristic load-bearing capacity of one cut of one fastener, Relation (1); *F*_v,E_ is a force acting on one cut of one fastener; and “Use” is the percentage utilization of the connection.

**Table 7:** Comparison of results of individual computational approaches.

Method of Calculation	Force Causing Collapse *F* (kN)	Bending Moment Causing Collapse (kNm)	Multiplier *M* (−)
Standard calculation EC5 [40]	132.00	198.00	-
*ANSYS*^TM^—linear calculation	308.89	469.51	1.96
*ANSYS*^TM^—nonlinear calculation	157.94	240.07	1.20
Experimental test	150.40	225.60	1.14

**Table 8:** Load-bearing capacity of individual frame connections.

Test No.	Capacity Experimental Testing (kN)	Capacity Analytical Calculation (kN)	Ratio (−)
1	233.30	132.00	1.767
2	161.30	102.00	1.581
3	150.40	132.00	1.139
4	133.00	102.00	1.304

**Table 9:** Table of stiffness.

Frame Connection	Serviceability Limit State			Ultimate Limit State			Load Value (%)	Ratio (−)
	*F* (kN)	*K*_r_ (MNm/rad)	EC5	*F* (kN)	*K*_r_ (MNm/rad)	EC5		
Bolt	40.80	34.258	20.081	-	-	-	40	1.706
	-	-	-	56.44	20.338	13.387	90	1.519
Screws	52.49	36.902	22.552	-	-	-	40	1.636
	-	-	-	73.28	21.617	15.033	90	1.438

## Data Availability

Data are contained within the article.
